# The molecular basis of the anticancer effect of statins

**DOI:** 10.1038/s41598-024-71240-6

**Published:** 2024-08-31

**Authors:** Giovanni Buccioli, Carolina Testa, Emanuela Jacchetti, Pietro Pinoli, Stephana Carelli, Stefano Ceri, Manuela T. Raimondi

**Affiliations:** 1 Department of Chemistry, Materials and Chemical Engineering “Giulio Natta”, Politecnico di Milano, Milan, Italy; 2 Department of Electronics, Information and Bioengineering, Politecnico di Milano, Milan, Italy; 3 Center of Functional Genomics and Rare Diseases, Buzzi Children’s Hospital, Milan, Italy

**Keywords:** Statins, Drug repurposing, Synthetic lethality, Metastases, Big data, Biomedical engineering, Cancer therapy, Computational science

## Abstract

Statins, widely used cardiovascular drugs that lower cholesterol by inhibiting HMG-CoA reductase, have been increasingly recognized for their potential anticancer properties. This study elucidates the underlying mechanism, revealing that statins exploit Synthetic Lethality, a principle where the co-occurrence of two non-lethal events leads to cell death. Our computational analysis of approximately 37,000 SL pairs identified statins as potential drugs targeting genes involved in SL pairs with metastatic genes. In vitro validation on various cancer cell lines confirmed the anticancer efficacy of statins. This data-driven drug repurposing strategy provides a molecular basis for the anticancer effects of statins, offering translational opportunities in oncology.

## Introduction

Cancer is an astonishingly complex disease and continues to challenge medical research and therapeutic strategies, with 1,918,030 new cases and 609,360 cancer related deaths in 2022^1^. In spite of the considerable progresses achieved in cancer research, traditional treatments are still accompanied by well-known side effects and various drawbacks. For instance, surgery and radiotherapy, beyond their positive effects, often inadvertently accelerate tumor growth and invasion due to the body’s inflammatory response and the variable cellular susceptibility to radiation^2,3^. Meanwhile, chemotherapy, while effective in reducing primary tumor volume^4^, is frequently undermined by the emergence of resistance mutations in cancer cells^5^. This resistance often leads to the progression of tumors with the development of more aggressive phenotype^6^.

Metastatic tumors, due to their diffuse localization and acquired resistance to cytostatic and cytotoxic agents, are particularly challenging to treat. Despite the availability of over 200 approved anti-cancer drugs, none have proven effective in inhibiting or treating cancer metastasis^7^, underscoring the urgent need for innovative therapeutic regimens. In this scenario, immunotherapy has emerged as a promising approach, leveraging the body’s immune system to target cancer cells^8^. However, its efficacy in treating solid tumors has been limited due to their inherent complexity^9^. Furthermore, recent studies have identified instances of secondary malignant tumors following therapy with CAR-T cells^10^.

The newly emerging therapeutic approach based on Synthetic Lethality (SL) could offer an encouraging solution^11^. It hinges on the simultaneous suppression of two genes, leading to cellular lethality, while the inhibition of each gene in isolation remains a non-lethal event^12^. It thus selectively targets cancer cells with specific mutations, sparing healthy cells and reducing toxicity^11^. This approach could be particularly potent for metastatic tumors, which tend to harbor more genetic mutations^13^, thereby potentially exerting anti-metastatic effects. SL therapy, therefore, holds the promise of providing a more effective, personalized treatment with a possible limitation of side effects^14^.

SL therapeutics, despite their promise, have encountered obstacles in clinical translation^15^. The challenges are twofold: not only is the laboratory-identification of robust SL gene pairs from the myriad possible combinations in a mammalian cell a complex task^15^, but the subsequent formulation and testing of a drug that effectively targets these identified genes also requires significant research investment^16^. Drug repositioning, the practice of repurposing existing drugs for new therapeutic applications, emerges as a promising strategy in the challenging landscape of drug development^17^. It offers reduced development timelines and financial burdens compared to traditional drug development^17^. Drug repurposing can be experimental-driven, often arising serendipitously^18^, or data-driven, a hypothesis-driven approach that uses big data to identify drugs against targets^19^. The latter transforms system biology data into predictions of druggable targets, ideally providing an FDA-approved compound with potential modulatory functions^19^. This approach requires accurate computational pipelines and algorithms for data integration and has been facilitated by the accumulation of high-throughput data and advances in computational and data sciences. In these circumstances, thanks to computer-aided approaches, it is possible to turn non-targeted (old) therapies into personalized treatments by selecting better responders’ patients^20^. Currently, there are approximately 2500 drugs that have received approval from the FDA^21^. Regrettably, a mere three drugs have been repurposed for cancer treatment: the Bacillus Calmette-Guerin vaccine for superficial bladder cancer, thalidomide for multiple myeloma, and propranolol for infantile hemangioma^22^. Furthermore, in the current scientific landscape, there is a stark absence of research that utilizes SL for the repositioning of drugs with anti-metastatic. This glaring omission not only underscores the urgency for innovative research but also highlights the potential for a paradigm shift in the treatment of metastatic diseases. The exploration of this uncharted territory could potentially herald a new era in cancer therapy, revolutionizing both the therapeutic approach and transforming patient outcomes.

Our research, integrating both experimental and computational studies, is aimed at developing a comprehensive approach to investigate repurposable drugs for the treatment of metastatic solid tumors, leveraging the concept of SL. This is achieved through the creation of integrated databases and computational analysis of diverse datasets, with the goal of identifying the most promising candidates for experimental validation. Among the top candidates, we selected Simvastatin and Lovastatin, members of the statin family, due to their widespread prescription^23^ and retrospective meta-analyses^24,25^ highlighting their antitumor effect. Our work represents a pioneering effort in integrating computational and experimental approaches to investigate repurposable drugs with anti-metastatic effect on metastatic solid tumors, leveraging the potential of SL, and ultimately demonstrating the antitumor therapeutic principle of statins.

## Method

### Data collection and integration

Our research is anchored in a dynamic computational framework that seamlessly integrates genes, drugs, and cancer types. This is not a static structure, but a vibrant network that intertwines five distinct categories of relationships, creating a holistic view of the complex interplay between these entities. We begin by exploring the associations between the metastatic phenotype of cancer and their corresponding gene conditions, with a particular focus on genes that, when deleted, are associated with metastases. This foundational knowledge guides us to the next phase: the identification of Synthetic Lethal (SL) gene pairs. Here, we search for pairs where one gene’s deletion is linked to a metastatic phenotype, while the other is not, thereby revealing potential genetic vulnerabilities in cancer cells. This understanding allows us to identify drugs that target these genes, opening the door to the development of effective treatments for specific cancer types. We place a special emphasis on repurposable drugs, those already approved for other indications, as this strategy can significantly accelerate the introduction of new treatments into clinical practice. These repurposable drugs, having undergone extensive testing, provide us with a wealth of safety and efficacy data, saving valuable time and resources in the drug development process. Finally, we explore the potential for synergy between these repurposable drugs and chemotherapeutic agents. The combination of two drugs can yield a comprehensive cytostatic and antimetastatic effect, halting the growth of cancer cells and preventing their spread to other parts of the body.

#### DataBases

1. cBioPortal^26^ has been employed for the identification of gene mutations implicated in metastases. Specifically, it has been done within the context of a pan-cancer metastatic solid tumor study^27^. This comprehensive study encompasses whole-exome and -transcriptome sequencing of 500 adult patients with metastatic solid tumors and primary normal pairs of diverse lineage and biopsy sites. Within this rich dataset, genes are validated as oncogenes through comparison with data from OncoKB^28^, helping the study to provide valuable insights into the gene’s expression status in metastatic scenarios. A modest increase in the tumor mutation burden was observed when metastatic cancers were contrasted with primary ones. This observation underscores the notion that the complete mutation spectra are likely to be formulated either preceding or during the advancement of primary cancer^29^.
2. SynLethDB^30^ houses all SL pairs discovered to date, collated through various investigative techniques. The database is a rich amalgamation of diverse sources, incorporating experimental data from biochemical assays, literature-derived information, publicly available datasets, computational predictions, and knowledge extracted from textual sources.
3. PanDrugs^31^ serves as a robust engine for prioritizing anticancer drug treatments based on individual multi-omics data. It is a culmination of data from an impressive 23 primary sources, resulting in a vast repository of 74,087 drug-target associations involving 4642 genes and 14,659 unique compounds. It provides comprehensive information on both the drug and the drug’s target gene; it also details the status of the drugs, including those in clinical trial, approved as antitumor, and approved for non-antitumor therapy.
4. DrugComb^32^ is a comprehensive drug sensitivity data repository and analysis portal. As an open-access, community-driven data portal, it accumulates, standardizes, and harmonizes the results of drug combination screening studies conducted across a diverse array of cancer cell lines. This database was selected to identify potential synergistic pairs composed of chemotherapeutic and repositioned drug.

#### Scores

Our selection of best candidates was guided by scores integrated into the aforementioned databases:

1. Synthetic Lethality Score (SLScore)^30^ from SynLethDB provides a detailed and quantitative view of synthetic lethality interactions, facilitating the discovery of potentially effective pharmacological targets for cancer treatment. A higher SLScore indicates a higher likelihood that the gene pair would be lethal if both genes are inactivated. This score is derived based on the annotated experimental methods from the evidence sources.
2. The Gene Score (GScore)^31^ from PanDrugs, ranging from 0 to 1, evaluates the biological relevance of the gene. It considers factors such as its essentiality, vulnerability, relevance in cancer, biological impact, frequency in genetic variation databases, and clinical implications. The GScore is updated regularly to incorporate the latest research findings, ensuring its relevance and accuracy.
3. The Drug Score (DScore)^31^ from PanDrug, spanning from $-1$ to 1, gauges the suitability of the drug based on factors such as drug-cancer type indications, clinical status, gene-drug relationships, support from curated databases, and collective gene impact.
4. The Zero Interaction Potency (ZIP) synergy score^32^, a key metric in DrugComb, provides a quantification of the degree to which the combined influence of two drugs exceeds the aggregate of their independent effects, under the presumption of non-interaction.

These scores are not opinion-based but are derived from robust methodologies, ensuring their reliability and consistency over time. They are continuously updated and refined as new data becomes available, ensuring that our candidates is always based on the most current and reliable information. In this way, the underlying methodologies and principles guiding the calculation of these scores remain consistent, ensuring their reliability and validity over time. This approach allows us to strike a balance between adaptability and consistency, ensuring that our research is both responsive to new developments and grounded in solid, reliable methods.

#### Pipeline

Building on the goal of finding a repurposable therapy that leverages the concept of SL, the inception of our study involved the identification of oncogenes that were deleted in metastatic conditions, achieved by querying the pan-cancer metastatic solid tumor study via cBioPortal. The gene selection was predicated on two specific criteria: a deleted expressive state in the metastatic phenotype and an oncogenic classification. The former was inferred from the “Variant Type” information, specifically the “DEL” term, indicative of deleted genes. The latter was facilitated by an integrated dataset with OncoKB in the study; the genes initially identified were cross-referenced with this dataset, selecting those that yielded a positive response to the “Is Cancer Gene (OncoKB)” query.

This curated gene pool of deleted oncogenes in metastases was subsequently cross-referenced with SL pairs within the SynLethDB database. To refine the selection process and mitigate the computational load, we considered only those pairs within the fourth quantile or above, revealing pairs with an SLScore exceeding 0.5. Notably, only one gene from each SL pair was required to be part of the previously curated pool.

**Fig. 1:**
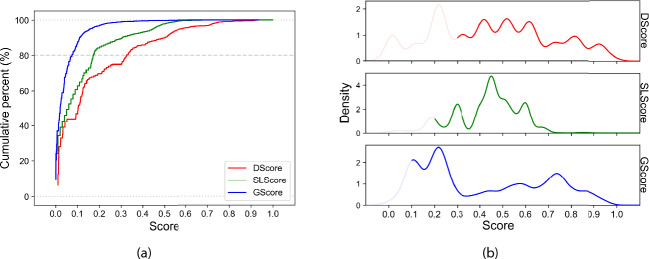
(**a**) Cumulative distribution curve of the three scores extracted for all possible drugs independently; lines represent the drug score (red), the SL score (green), and the gene score (blue). An 80% threshold has been set to identify the initial minimum significant threshold of scores for candidate filtering, according to Pareto principle. By intersecting the curves and the 80% cutoff, preliminary thresholds were identified at DScore > 0.3, SLScore > 0.2, GScore > 0.1 (**b**) Probability distribution curves for the three scores are delineated; lines signify the drug score (red), the SL score (green), and the gene score (blue). The probability distribution of scores is a tool in statistics and data analysis, offering valuable insights into variable understanding, hypothesis testing, and outcome prediction. The area under the probability density function curve between two points corresponds to the probability that the variable falls within that interval of score. The score corresponding to the first notable peak beyond the minimum threshold, determined by the individual score corresponding to a cumulative percent greater than 80%, is chosen for each score. Consequently, the final thresholds were determined to be DScore > 0.35, SLScore > 0.5, and GScore > 0.35.

The list of counterpart genes, corresponding to non-mutated genes, was then sought in PanDrugs to identify potential drug targets. Furthermore, we conducted an exhaustive screening of the database to identify pharmaceutical agents suitable for repurposing. Since our objective was to develop a comprehensive therapy that combines a repurposed anti-metastatic drug with a conventional chemotherapy, a first selection was performed by excluding chemotherapeutics as indicated by the “Therapy” entry. Hence, to finally identify repurposable drugs, we utilized the information obtained from PanDrugs in “Status Description”: drugs that presented the term “Cancer Clinical Trials and approved for other pathologies” were considered repurposable.

Among the drugs identified, a further selection was made to identify synergistic combinations with a conventional chemotherapeutic. This research is of considerable importance as the combination with a chemotherapeutic protocol could potentially fast-track its implementation in a clinical setting. This was accomplished via DrugComb, identifying previously discarded chemotherapeutics in combination with repurposable drugs exhibiting a ZIP score exceeding 0.

In our quest to identify the most promising candidates, we employed a tripartite scoring system encompassing drug, synthetic lethality, and gene scores, in according with other example in literature^12^. An initial filtering was conducted using a common principle for identifying the threshold of all scores, namely the 80% of the cumulative percentage, a widely accepted benchmark rooted in the Pareto principle or the 80/20 rule^33^. The calculation of cumulative percentages provides valuable insights into score distribution, trend identification, and impact evaluation, highlighting the relative positioning of specific score points. The preliminary thresholds for selecting the scores corresponding to cumulative percentages above 80% were identified independently for each curve, specifically at 0.3 for the Drug Score (DScore), 0.2 for Synthetic Lethality pairs (SLScore), and 0.1 for the Gene Score (GScore) (Fig. 1a). Through this filtering process, the number of candidates is reduced by 35%. However, given the extensive pool of remaining candidates even after the initial filtering, a secondary, more stringent filtering was deemed necessary. This involved identifying significant thresholds based on each specific score’s probability distribution. The probability distribution of scores is a tool in statistics and data analysis, offering valuable insights into variable understanding, hypothesis testing, and outcome prediction. Taking as a starting point the three scores highlighted by the cumulative percentages filtering, we identified and set the second threshold nearby the first significant peaks after the previous minimum scores, representing the highest likelihood of encountering candidates meeting our filtering criteria. The area under the probability density function curve between two points corresponds to the probability of the variable falling within that score interval. Consequently, the final thresholds were determined to be DScore > 0.35, SLScore > 0.5, and GScore > 0.35, effectively narrowing down the pool of potential candidates (Fig. 1b). This rigorous, two-tiered approach ensures a comprehensive and precise identification of the most promising candidates. In the process of calculating the minimum scores, we made a conscious decision to leverage the full potential of the databases, hence, in the case of SynLethDB, we did not restrict it to the fourth quartile. This was primarily because such a limitation would not have facilitated an accurate distribution of probabilities and cumulative percentages. In line with this, the limit determined based on the SLScore was found to be equivalent to the score pinpointed at the fourth quartile.

### Cell cultures and reagents

#### Cell lines, culture, and reagents

The MDA-MB-231 and HeLa cell (ATCC, US), a breast adenocarcinoma cell line and a cervical cancer respectively, were maintained in Dulbecco’s Modified Eagle Medium (DMEM, Euroclone, Italy). The HCC1937 (CLS, Germany), a breast ductal carcinoma cell line, and OVPA8 (DSMZ, Germany), a high-grade serous ovarian adenocarcinoma, were cultured in RPMI 1640 medium (Euroclone, Italy). Both media were supplemented with 10% fetal bovine serum (FBS, Euroclone, Italy), 1% l-glutamine (Euroclone, Italy), and 1% penicillin-streptomycin (Euroclone, Italy). All cell lines were incubated at 37 $^\circ$C in a humidified atmosphere containing 5% CO$_{2}$. Table 1 provides a comprehensive catalogue and elucidation of the cell lines, specifically focusing on their respective states of gene expression of interest. Lovastatin (Mevinolin, MedChemExpress, US) and Simvastatin (MK733, MedChemExpress, US), members of the lipophilic group of statins, were prepared as drug solutions in Dimethyl sulfoxide (DMSO, PanReac AppliChem, Italy) and stored at $-80^\circ$C.

**Table 1:** Overview of the cell types utilized in the experimental validation and their corresponding gene expression related to the pharmacological test of Simvastatin and Lovastatin (wt: Wild-Type, mut: Mutated).

MDA-MB-231	HeLa	HCC1937	OVPA8
BRCA1-wt	BRCA1-wt	BRCA1-mut	BRCA1-mut
KRAS-mut	KRAS-wt	KRAS-wt	KRAS-wt

#### Cell viability assay

To assess the efficacy of the drugs on cancer cell lines with various mutations, we designed experiments to draw viability curves and determine the Half-maximal inhibitory concentration (IC50) for each drug on each cell line. The Cell Counting Kit-8 (CCK8, Prodotti Gianni, Italy) assay, a reliable method for gauging cellular metabolic activity as a proxy for cell viability, was employed. Initially, 2000 cells per well were seeded in 96-well plates and incubated overnight in 100 µL of medium. Subsequently, cells were exposed to a gradient of drug concentrations for 72 h, ranging from 0.01 to 40 µM for Simvastatin, and 1 pM to 100 µM for Lovastatin. Notably, the volume of drug diluted in DMSO added to the cell culture was consistently kept below 0.05% in each experiment to minimize its potential influence on the cellular response to the drugs. After 72 h, the CCK8 was introduced at a 1:10 dilution in standard culture medium, and incubated for 1–3 h at 37 $^\circ$C, until the solution turned orange. Absorbance at 450 nm was then measured using a Tecan (Infinite M200PRO, Switzerland) microplate reader.

#### Statistical analysis

Cell viability data to determine the IC50 were analyzed using GraphPad Prism (Version 9.5.1). The data underwent normalization, with cells treated solely with DMSO established as the benchmark for 100% viability, and wells containing only medium and CCK8 designated as the 0% reference point. The concentrations were converted into logarithmic form to facilitate the construction of the viability curve. This was accomplished by generating a non-linear regression curve that provided an optimal fit with the data, thereby enabling a robust analysis of cell viability across varying drug concentrations. For the appraisal of the congruence between the regression curves and the empirical data, only those instances where the R^2^ values greater than 0.8 were factored into the analysis. Each experimental procedure was executed with a baseline of four replicates. The outcomes are articulated as mean values, augmented by their respective standard deviations.

## Results

### Data-driven identification of the best repurposable candidates

**Fig. 2:**
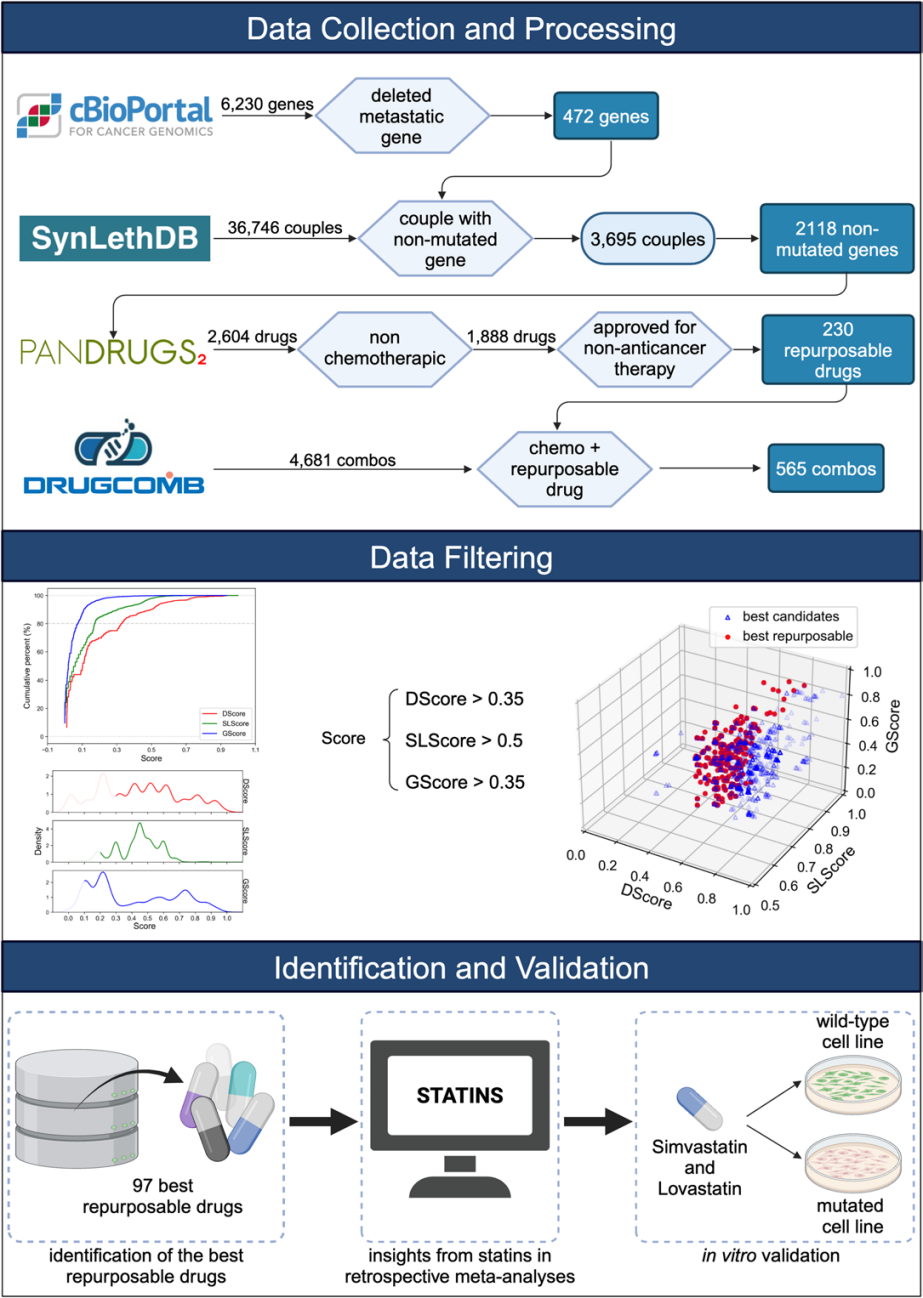
Graphical scheme of the three stages of our research approach: (i) *Data Collection and Processing*: Three types of entities (genes, drugs, and cancer types) are gathered and scrutinized from databases to explore the landscape of anti-metastatic repurposable drugs via SL. The dataset encompasses five types of relationships (metastatic gene, SL gene pair, drug–gene relationship, relationships between drugs and their approved indications, and synergistic drug combination), providing the computational groundwork for drug repositioning. (ii) *Data Filtering*: The dataset is sorted based on GScore, DScore, and SLScore values. The most promising candidates are pinpointed through cumulative percentages and alignment with the density distribution. Optimal scores are identified as GScore > 0.35, SLScore > 0.5, and DScore > 0.35. Within the pool of best candidates, a focused search was conducted to identify those with repurposable potential. (iii) *Identification and Validation*: Among the best repurposable candidates, statins have demonstrated antitumor activity in accordance with retrospective meta-analyses. The in vitro experimental validation is carried out with cell lines presenting necessary mutations for susceptibility and wild type cell lines as a negative control.

**Fig. 3:**
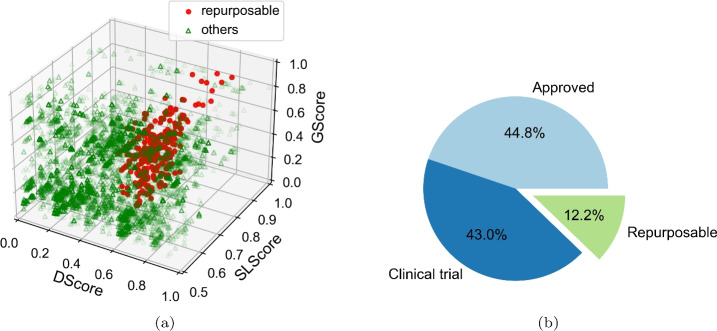
Collection and processing of candidates to identify the repurposable drugs usable for the anticancer Synthetic Lethality approach. (**a**) The three-dimensional representation provides insight into the targeting of non-mutated genes in a Synthetic Lethality association with a deleted metastatic gene. The green triangles signify all the discerned drugs that target non-deleted genes in an SL pair with deleted metastatic genes, and are non-chemotherapeutic, totaling 1888. Among these, 230 candidates are repurposable (red dot). (**b**) The pie chart provides a visual representation of the distribution of the 1888 drugs we identified, grouped according to their status. Specifically, 44.8% of these drugs are already approved for cancer treatment, 43.0% are in clinical trial phase, while 12.2% encompass commercially available drugs that are used for non-cancer related treatments but have potential for repurposing in cancer therapy.

The comprehensive framework of our study, as illustrated in Fig. 2, encapsulates the methodology employed to discover repurposable drugs that inhibit genes forming Synthetic Lethality (SL) pairs with deleted metastatic genes, followed by subsequent in vitro validation. Oncogenes associated with metastasis were curated from cBioPortal, resulting in a refined subset of 472 genes. We then sought SL partners of these genes within the SynLethDB database, distilling our initial pool of 36,746 pairs down to a final selection of 3695 pairs above the fourth quantile, extrapolating 2118 targetable non-mutated genes. The subsequent phase involved identifying agents that can target these selected non-mutated genes, using associations found within PanDrugs. Our investigation uncovered 2594 pharmaceutical compounds that target these genes. We focused on non-chemotherapeutic agents, resulting in a selection of 1888 compounds. Out of these, 230 were repurposable drugs (Fig. 3a). Thus, 12.2%, of the drugs under investigation are currently approved for therapies not related to cancer (Fig. 3b). However, these drugs exhibit potential for repurposing in oncological treatments that leverage the concept of SL.

**Fig. 4:**
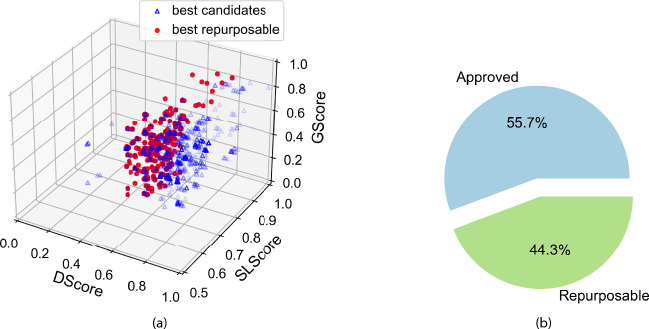
Graphical description of the filtering process applied during the identification of best candidates. (**a**) Through the implementation of statistically determined scoring thresholds (DScore > 0.35, SLScore > 0.5, GScore > 0.35), we have identified a subset of candidates exhibiting superior promise. This has resulted in a pool of 219 best candidates, represented by blue triangles. Within this pool, the number of repurposable drugs is further refined to 97, distinctly visualized as red dots. (**b**) Among the 219 best candidates, 55.7% have already received approval for use in cancer treatment, while 44.3% are commercially available drugs currently used for non-cancer treatments but show potential for repurposing in cancer therapy.

We further analysed all potential candidates within our dataset, categorizing them based on their respective GScore, DScore, and SLScore values. Consequently, out of initial candidates, 219 exhibited more compelling characteristics (listed in Supplementary Table 1), and of these, 97 were repurposable (Fig. 4a, listed in Supplementary Table 2). Notably, our analysis identified approximately 44.3% of the top candidates as potentially repurposable pharmaceuticals (Fig. 4b). This highlights the untapped potential within the existing pharmacopeia and signals a paradigm shift towards repurposing existing compounds for novel therapeutic applications.

Among the repurposable pharmaceutical agents that exploit the SL principle, we made thoughtful selections for subsequent experimental validation of data-driven results. Statins emerged as the most compelling options due to the presence in literature of retrospective meta-analyses and experiments that demonstrate their antitumor activity without being able to explain the therapeutic principle beyond this activity^24,34–37^. Statins target genes HDAC2 and HMGCR which form SL pairs with BRCA1 and KRAS respectively (Table 2). Among the statin family identified through computational analysis, we chose Simvastatin and Lovastatine as the most clinically relevant candidates for experimental testing.

**Table 2:** The two tables show the computational findings pertaining to the (**a**) HDAC2 and (**b**) HMGCR genes, and their corresponding Synthetic Lethality pairments with deleted metastatic genes, which are inhibited by drugs of the statin family (Atorvastatin, Lovastatin, Simvastatin, Pravastatin). The SL pairs that we selected for experimental validation are highlighted in bold.

Mutated gene	Target gene
(a)	
BRAF	HDAC2
**BRCA1**	**HDAC2**
SMARC4	HDAC2
VHL	HDAC2
(b)	
ATM	HMGCR
CDK12	HMGCR
ERBB3	HMGCR
FGFR4	HMGCR
**KRAS**	**HMGCR**
PRKDC	HMGCR

As an extension to our primary focus, we also conducted an inquiry to identify chemotherapeutic agents that could synergize with statins. This inquiry is deemed essential, as the integration with a chemotherapeutic agent could potentially facilitate the rapid deployment of the repurposable drug identified here in a clinical setting. From a pool of 4691 potential synergistic drug pairs, we discovered 565 couples consisting of chemotherapeutic agents paired with repurposed drugs. Notably, statins are paired with the chemotherapy Temozolomide, an oral alkylating agent.

### In vitro validation of Simvastatin and Lovastatin as anticancer agents

Statins are widely utilized in the management of cholesterol levels and cardiovascular diseases^38^; they have emerged from our computational analysis as a prime candidate for experimental validation. This selection is underscored by retrospective meta-analyses on large patient cohorts that have revealed an unexpected antitumor potential of statins^24,39^. We speculated that cell lines possessing Synthetic Lethality (SL) pair mutations with genes susceptible to statins, may possess a heightened sensitivity to this drug (Table 3). On the other hand, cell lines devoid of these mutations are anticipated to exhibit resistance to drug treatment.

**Table 3:** Sensitivity to Simvastatin and Lovastatin of cell lines harboring genes that are mutated and in Synthetic Lethality pairs. The table reports the specific mutated genes for each cell lines treated with Simvastatin or Lovastatin, along with the corresponding IC50 values identified.

Cancer type	Cell line	Mutated gene	Target gene	Simvastatin IC50 (µM)	Lovastatin IC50 (nM)
Breast	MDA-MB-231	KRAS	HMGCR	2.108	9.5
Breast	HCC1937	BRCA1	HDAC2	20.94	8700
Ovarian	OVPA8	BRCA1	HDAC2	8	522
Cervix	HeLa	–	–	No effect	No effect

**Fig. 5:**
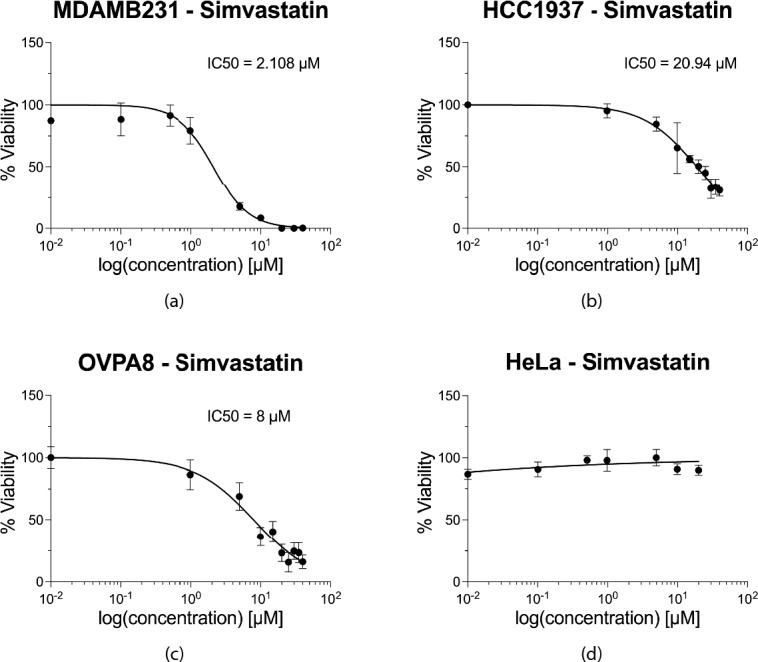
Validation of the Synthetic Lethality hypothesis with Simvastatin. Half-maximal inhibitory concentration (IC50) is determined for different cancer cell lines, after 72 h of treatment with Simvastatin. (**a**) The MDA-MB-231 cell line, harboring a mutated KRAS gene that forms an SL pair with HMGCR, demonstrated an IC50 value of 2.108 µM, R^2^ = 0.9554. (**b**) The HCC1937 cell line, harboring a mutated BRCA1 gene that forms an SL pair with HDAC2, demonstrated an IC50 value of 20.94 µM, R^2^ = 0.8715. (**c**) The OVPA8 cell line, harboring a mutated BRCA1 gene that forms an SL pair with HDAC2, demonstrated an IC50 value of 8 µM, R^2^ = 0.8427. (**d**) The HeLa cell line, devoid of mutations that form SL pairs with the target genes of Simvastatin, remains unaffected across all tested Simvastatin concentrations.

**Fig. 6:**
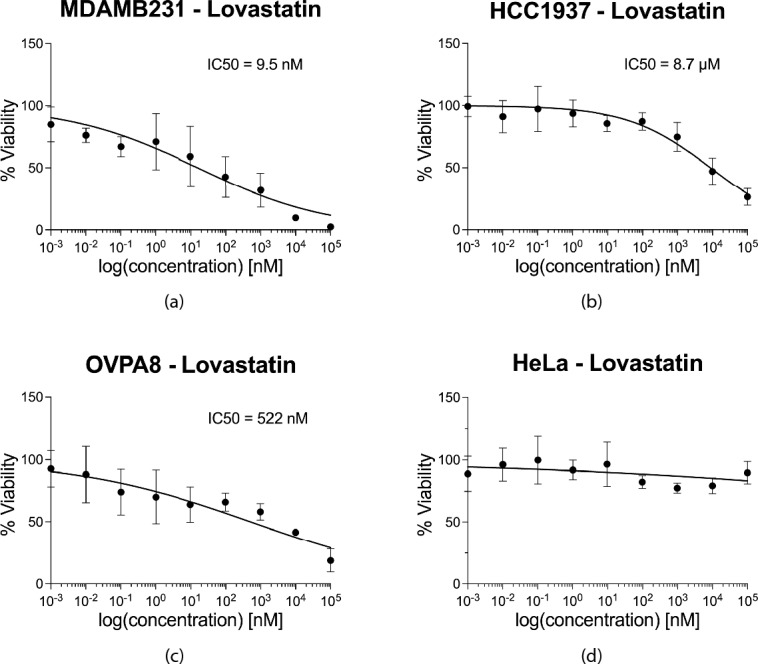
Lovastatin validation of the Synthetic Lethality hypothesis. Half-maximal inhibitory concentration (IC50) is determined for different cancer cell lines, after 72 h of treatment with Lovastatin. (**a**) The MDA-MB-231 cell line, harboring a mutated KRAS gene that forms an SL pair with HMGCR, demonstrated an IC50 value of 9.5 nM, R^2^ = 0.8. (**b**) The HCC1937 cell line, harboring a mutated BRCA1 gene that forms an SL pair with HDAC2, demonstrated an IC50 value of 8.7 µM, R^2^ = 0.82. (**c**) The OVPA8 cell line, harboring a mutated BRCA1 gene that forms an SL pair with HDAC2, demonstrated an IC50 value of 522 nM, R^2^ = 0.78. (**d**) The HeLa cell line, devoid of mutations that form SL pairs with the target genes of Lovastain, remains unaffected across all tested Lovastatin concentrations.

To validate this hypothesis, we performed drug tests to determine the half-maximal inhibitory concentration (IC50) of Simvastatin and Lovastatin for several cell lines (MDA-MB-231, HCC1937, OVPA8 and HeLa; Table 1). Cell lines harboring mutations susceptible to Simvastatin demonstrated a significant drug response with dosages varying in the range of tens of µM (from 0.01 to 40 µM) (Fig. 5). On the other hand, the same cell lines show greater sensitity to Lovastatin, in the order of some nM (Fig. 6). Specifically, the IC50 for Simvastatin and Lovastatin were respectively equal to 2.108 µM and 9.5 nM for MDA-MB-231 breast cancer-derived cells, 20.94 µM and 8.7 µM for HCC1937 breast cancer cells, 8 µM and 522 nM for OVPA8 ovarian cancer cell line. In contrast, coherently with our hypothesis, HeLa cells, which do not carry mutations in the SL genes defined here, did not exhibit any susceptibility to either drug, although the drugs may have a cytotoxic effect at exposure times longer than 72 h and/or at higher concentrations. Thus, the experimental data validated our hypothesis, firmly establishing statins as antitumor agents, of which the activity is unequivocally due to the phenomenon of SL.

## Discussion

**Fig. 7:**
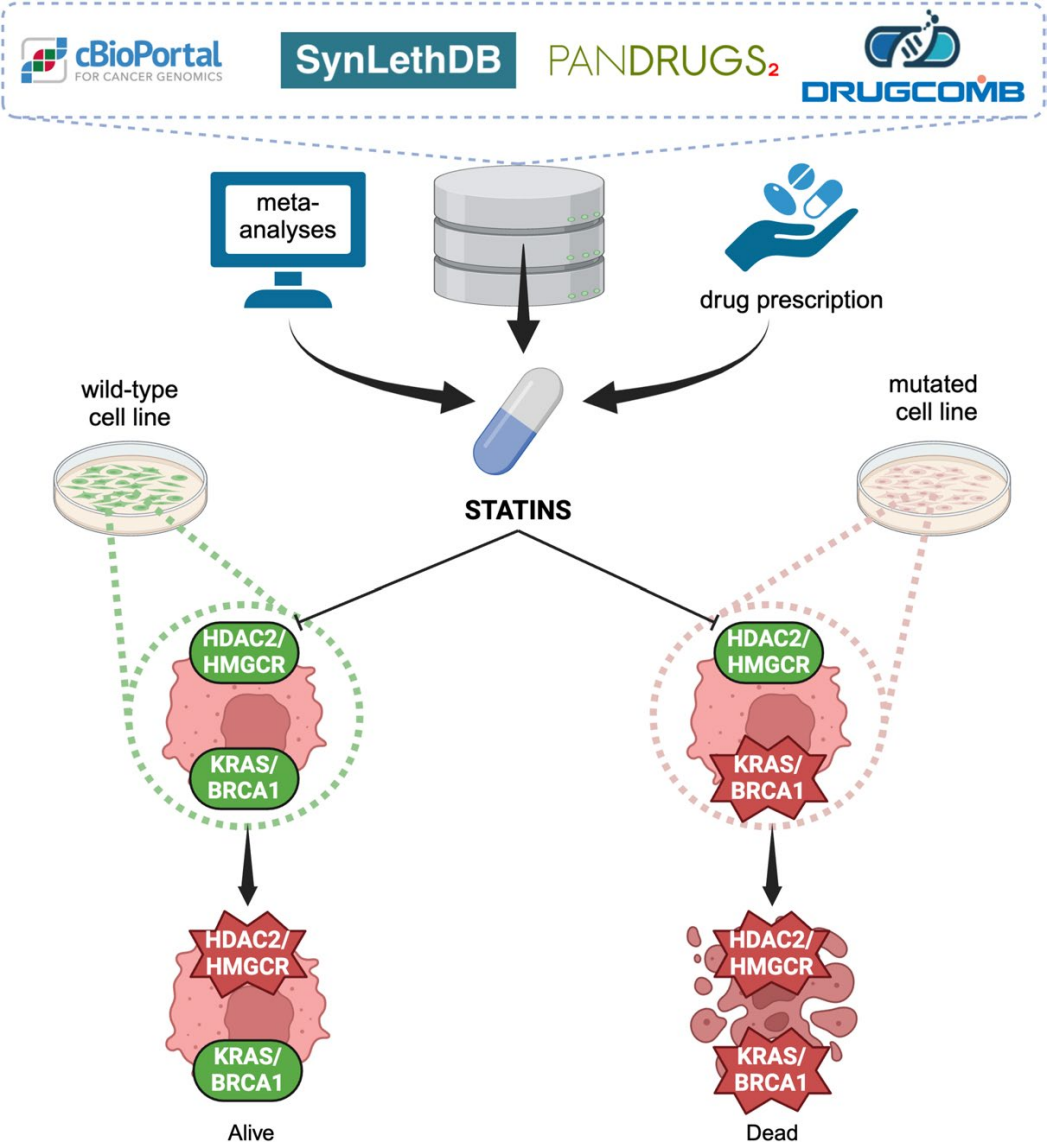
Simvastatin and Lovastatin, selected from our data-driven analysis and substantiated by retrospective meta-analyses and extensive current prescriptions data, have demonstrated their antitumor activity rooted in Synthetic Lethality through experimental validation. This compelling evidence irrefutably establishes its therapeutic principle of statins, marking a significant stride in the realm of personalized medicine based on data-driven repurposed drugs.

Computational methodologies are progressively being embraced in the scientific community as a valuable adjunct to traditional experimental procedures, with the objective of expediting discovery processes and unveiling novel targets^40^. Several groups have embarked on computational explorations to pinpoint gene pairs demonstrating Synthetic Lethality (SL)^12,41^, with a subset harnessing the potential of machine learning to uncover new SL couples^42^. Simultaneously, others have delved into databases in pursuit of drugs amenable to repurposing^19^. Yet, no one has proposed a data-driven approach akin to ours, where we have identified repurposable drugs that target genes exhibiting SL when coupled with deleted metastatic genes. This strategy may lead to new antimetastatic therapeutic approach, surpassing the limitations of conventional treatments. Moreover, SL aligns well with personalized medicine, allowing for treatments to be tailor-made based on each patient’s individual mutation profile, resulting in a highly effective therapy with a reduced risk of toxicity^14^. Furthermore, repurposed drugs offer potential savings in terms of time and money, and they have a higher likelihood of obtaining regulatory approval^17^. Through this computational approach, we discovered that 12.2% of all identified drugs could potentially be repurposed. Although this proportion might appear modest, it does not encompass all potential drugs that have yet to be reevaluated for alternative therapeutic applications. To ensure the validity and precision of our computational results, we imposed stringent thresholds, leading to a refined selection of 219 potential drug candidates. Remarkably, our analysis revealed that approximately 44.3% of these top candidates could be considered for pharmaceutical repurposing. Moreover, integrating various databases, we achieved results absent in the literature to date, such as the explanation of the antitumor activity of hydrophobic statins beyond their classical function in cholesterol biosynthesis. Reviews of the existing literature on statin experimentation reveals a multitude of secondary and pleiotropic effects identified by various researchers^35–37^. However, the elucidation of a definitive therapeutic principle within these studies remains elusive. Despite this, retrospective meta-analyses have corroborated their effectiveness in preventing and treating both breast and ovarian metastatic tumors^24,25,39,43,44^. In this work, we interpret the anticancer mechanism of action of statins with SL analysis. Among the statins identified computationally, Simvastatin and Lovastatin were selected for experimental validation (Fig. 7). One of the genes targeted by statins is HMGCR, responsible for the inhibition of mevalonate pathway and prenylation^45^. Our computational analysis underscores how statins-induced inhibition of protein prenylation acts synergistically with other cancer-related genetic alterations, primarily KRAS, leading to SL. In the MDA-MB-231 cancer cell line, already characterized by KRAS mutations, a heightened susceptibility and subsequent mortality is observed in response to both Lovastatin or Simvastatin treatments. On the other hand, the HeLa cell line, which lacks mutations able to couple in SL pairs with the target genes of Simvastatin and Lovastatin, exhibits no changes across all examined drug concentrations. This underscores the antitumor efficacy of statins only in mutated cells. Intriguingly, the computational results have unveiled an unexpected interaction between BRCA1 and HDAC2, offering a novel perspective on the potential anticancer mechanism of statins. Specifically, statins induces SL in cancer cells with BRCA1 mutations when coupled with the inhibition of HDAC2, a histone deacetylase involved in epigenetic regulation^46^. BRCA1, a tumor suppressor gene implicated in DNA repair processes^47^, is frequently mutated in various cancers, particularly breast and ovarian cancers^48^. The connection between BRCA1 and HDAC2 suggests a delicate balance in maintaining genomic integrity. Consequently, statins, by targeting HDAC2 in BRCA1-deficient cells, exacerbate the underlying genomic instability, resulting in SL. Pharmacological assays of Simvastatin and Lovastatin, conducted in vitro on the HCC1937 and OVPA8 cell lines, which are characterized by a BRCA1 deletion, revealed a pronounced drug sensitivity. This observation further corroborates the computational findings previously obtained.

In the realm of breast cancer research, the mutation frequency of the BRCA1 gene exhibits a broad spectrum, ranging from 1.8 to 36.9%, contingent upon the specific tumor subtype under consideration^49^. A noteworthy observation is the elevated mutation rate of up to 15.4% in triple-negative breast cancer, a scenario that is often concomitant with an aggressive tumor stage^49^. In a parallel context, ovarian cancer patients demonstrate a similar proportion (15.4%) of BRCA1 mutations^50^. The mutation landscape of the KRAS gene, on the other hand, is characterized by a considerable degree of variability, influenced by factors such as the primary tumor site and its stage^51^. In colon cancer, for instance, the mutation frequency averages at 39.3%, with a pronounced escalation to 76.6% in the case of metastatic solid tumors^51^. A stark contrast is observed in triple-negative breast cancer, where a substantial 65% are found to harbor a KRAS mutation^52^. In light of the findings, it can be inferred that the patient population poised to benefit from statin therapy is substantial. This is particularly pertinent for patients grappling with metastatic tumors, who are often confronted with a paucity of efficacious therapeutic options and survival timelines that starkly contrast with the pace of research advancements.

In a correspondence to the editors of the British Journal of Clinical Pharmacology, Björkhem-Bergman et al.^53^ expressed concerns regarding the high concentrations of statins observed in vitro for cancer treatment, arguing that these levels are not negligible in a clinical setting. However, it is important to note that their literature review primarily focused on the pleiotropic effects of statins, which necessitate high concentration of the drugs. Our findings suggest that the IC50 values we obtained are compatible with clinical applications, especially in the case of Lovastatin, which exhibits lower effective concentrations. Jisng et al.^54^ conducted tests on various statins across several cell lines, and reported in vitro IC50 values between 0.2 and 70 µM in breast cancer cell line. When converted into standard doses, these values approximatein the order of milligrams. In clinical settings, the maximum dosage of Lovastatin in conventional applications is of 80 mg^55^, whereas for Simvastatin is of 40 mg^56^. Hence, the dosages of statins tested for treating specific tumors are of the same order of magnitude as those currently used in conventional anti-cholesterol therapy. In conclusion, the exploitation of SL allows for a highly specific, targeted cancer therapy. This approach not only reduces the required dosage while maintaining therapeutic efficacy but also repurposes a drug already in widespread clinical use, thereby cutting down on development time and costs.

## Conclusion

In our study, we employed a comprehensive data analysis approach to identify the most promising drug candidates that target non-mutated genes involved in Synthetic Lethality (SL) pairs with deleted metastatic genes. This analysis highlighted the BRCA1–HDAC2 and KRAS–HMGCR pairs, offering valuable insights into potential vulnerabilities in cancer cells that could be exploited by Simvastatin and Lovastatin. These findings open new avenues for personalized treatment strategies, particularly for patients with BRCA1-mutated or KRAS-mutated cancers. To further substantiate our findings, future work will involve testing additional cell lines and employing vectors as negative and/or positive controls. An intriguing prospect would be to examine the computationally identified synergy between statins and temozolomide, with the aim of developing a cytotoxic and anti-metastatic therapy that could be swiftly introduced into clinical practice. The potential of our approach lies in its ability to expedite the drug development process by repurposing already approved drugs, thereby reducing associated costs and risks. This strategy not only enhances the efficiency of precision oncology but also holds promise for improving patient outcomes.

### Supplementary Information

Supplementary Table 1.

Supplementary Table 2.

## Data Availability

The datasets and the code used and/or analysed during the current study available from the corresponding author on reasonable request. The data that support the findings of this study are provided within the supplementary information files. The databases used and/or analysed during the study are available on the corresponding web page (the corresponding link is present in the manuscript).
